# Genomic Breeding for Diameter Growth and Tolerance to *Leptocybe* Gall Wasp and *Botryosphaeria*/*Teratosphaeria* Fungal Disease Complex in *Eucalyptus grandis*

**DOI:** 10.3389/fpls.2021.638969

**Published:** 2021-02-26

**Authors:** Makobatjatji M. Mphahlele, Fikret Isik, Gary R. Hodge, Alexander A. Myburg

**Affiliations:** ^1^Mondi Forests, Research and Development Department, Trahar Technology Centre – TTC, Hilton, South Africa; ^2^Department of Biochemistry, Genetics and Microbiology, Forestry and Agricultural Biotechnology Institute, University of Pretoria, Pretoria, South Africa; ^3^Department of Forestry and Environmental Resources, North Carolina State University, Raleigh, NC, United States; ^4^Camcore, North Carolina State University, Raleigh, NC, United States

**Keywords:** ssGBLUP, genetic correlation, *Eucalyptus grandis*, *Leptocybe invasa*, *Botryosphaeria dothidea*, *Teratosphaeria zuluensis*

## Abstract

*Eucalyptus grandis* is one of the most important species for hardwood plantation forestry around the world. At present, its commercial deployment is in decline because of pests and pathogens such as *Leptocybe invasa* gall wasp (*Lepto*), and often co-occurring fungal stem diseases such as *Botryosphaeria dothidea* and *Teratosphaeria zuluensis* (*BotryoTera*). This study analyzed *Lepto*, *BotryoTera*, and stem diameter growth in an *E. grandis* multi-environmental, genetic trial. The study was established in three subtropical environments. Diameter growth and *BotryoTera* incidence scores were assessed on 3,334 trees, and *Lepto* incidence was assessed on 4,463 trees from 95 half-sib families. Using the *Eucalyptus* EUChip60K SNP chip, a subset of 964 trees from 93 half-sib families were genotyped with 14,347 informative SNP markers. We employed single-step genomic BLUP (ssGBLUP) to estimate genetic parameters in the genetic trial. Diameter and *Lepto* tolerance showed a positive genetic correlation (0.78), while *BotryoTera* tolerance had a negative genetic correlation with diameter growth (−0.38). The expected genetic gains for diameter growth and *Lepto* and *BotryoTera* tolerance were 12.4, 10, and −3.4%, respectively. We propose a genomic selection breeding strategy for *E. grandis* that addresses some of the present population structure problems.

## Introduction

Fast-growing plantation forests are essential to the pulp, paper, and timber industries and the emerging biorefinery and biomaterials industries (Perlack et al., 2005; Cetinkol et al., 2012; Devappa et al., 2015; Stafford et al., 2020). The sustainability of many of these industries is dependent on woody biomass from plantation-grown *Eucalyptus* trees. *Eucalyptus* species are adaptable, fast-growing, generally resilient to pests and pathogens, and have the desired wood qualities for diverse wood products (Malan, 1993; Stafford et al., 2020). Volume growth and wood density are essential measures for forest plantation productivity (Raymond, 2002). However, pest and pathogen challenges have increased in severity in the past decades, posing a significant risk to *Eucalyptus* plantation forestry productivity and sustainability in subtropical regions (Wingfield et al., 2015). How to ensure continued genetic gains for volume growth in the presence of severe pest and pathogen challenges has become an essential question for plantation species such as *Eucalyptus grandis*.

*Leptocybe invasa* Fisher & La Salle is one of the most damaging insect pests of *Eucalyptus* species that affects growth by forming galls on leaves and leaf petioles. The insect is native to Queensland, Australia, known as the Blue Gum Chalcid wasp (Hymenoptera: Eupholidea). It has spread across the globe, infesting a wide range of commercially grown *Eucalyptus* species and their hybrids, resulting in severe losses in young plantations and nursery seedlings (Mendel et al., 2004; Nyeko et al., 2010; Chang et al., 2012; da Silva et al., 2020). First reported in the Mediterranean Basin and the Middle East in 2000 (Viggiani et al., 2000; Mendel et al., 2004), *L. invasa* subsequently spread throughout countries in Africa, America, and Asia (Nyeko, 2005; Wiley and Skelly, 2008; Prabhu, 2010; Zhu et al., 2012). Two parasitoid species of *L. invasa* from Australia, *Quadrastichus mendeli* and *Selitrichodes kryceri*, were deployed as biological controls to manage severe infestation levels in *Eucalyptus* plantations in Israel (Kim et al., 2008). Tracking the introduction of *L. invasa* in South Africa, *Q. mendeli* was recently discovered, and the biological control potential of *L. invasa* in South African *Eucalyptus* plantations was investigated (Bush et al., 2018). Another recently discovered parasitoid species of *L. invasa* from Australia, *S. neseri*, was described and investigated for its parasitism rates in South Africa, ranging from 9.7 to 71.8% (Dittrich-Schroder et al., 2014).

Resistance-linked DNA markers for molecular breeding is an alternative strategy to manage pest challenges. Towards this, simple sequence repeat (SSR) markers have been identified that jointly explained 3–37% of the variation of resistance in *E. grandis* and were validated in *E. tereticornis* explaining 24–48% of the variation of resistance (Zhang et al., 2018). Due to the significant variation that exists within and between *Eucalyptus* species, there is opportunity to breed for *L. invasa* tolerance (Mendel et al., 2004; Thu et al., 2009; Durand et al., 2011; Sangtongpraow et al., 2011; Dittrich-Schroder et al., 2012; Nugnes et al., 2015; Zheng et al., 2016). A recent genome-wide association study in an *E. grandis* breeding population identified candidate genomic regions on chromosomes 3, 7, and 8 that contained putative candidate genes for tolerance. These candidate genomic regions explained ∼17.6% of the total phenotypic variation of *L. invasa* tolerance (Mhoswa et al., 2020).

*Teratosphaeria zuluensis*, a fungal pathogen that causes stem canker, previously known as *Coniothyrium* canker, is a devastating stem disease of *Eucalyptus* species and is one of the most severe pathogens of plantation-grown *Eucalyptus* spp. (Wingfield et al., 1996; Crous et al., 2009; Aylward et al., 2019). It was first recognized in South Africa in 1989 and described in 1996 (Wingfield et al., 1996). *T. zuluensis* has been reported on *Eucalyptus* spp. in Malawi, Mozambique and Zambia (Jimu et al., 2015), Hawaii (Cortinas et al., 2004), Ethiopia (Gezahgne et al., 2003), and Argentina and Vietnam (Gezahgne et al., 2004b). Infections from *T. zuluensis* results in necrotic spots on green branches and the main stem, giving a “cat-eye” appearance that develops into large cankers on susceptible trees. *T. zuluensis* infection reduces wood quality by penetrating the cambium to form black kino filled pockets and may lead to tree death (Wingfield et al., 1996; Gezahgne et al., 2003).

*Botryosphaeria dothidea* is also a devastating fungal pathogen of eucalypt species affecting the stem. *B. dothidea* is known to have endophytic characteristics with instances of opportunistic latent infections (Smith et al., 1996; Slippers et al., 2009). Species of the *Botryosphaeriaceae* family infect plants via natural apertures (Bihon et al., 2011) and wounding (Epstein et al., 2008). *B. dothidea* infection results in longitudinal cracks that penetrate the bark into the xylem forming kino pockets in the wood, and stem cankers and tip dieback (Smith et al., 1994). It infects eucalypts in many countries including the Congo (Roux et al., 2000), Australia (Burgess et al., 2019), South Africa (Smith et al., 1994), Ethiopia (Gezahgne et al., 2004a), Venezuela (Mohali et al., 2007), Colombia (Rodas et al., 2009), Uruguay (Perez et al., 2008), and China (Chen et al., 2011). Field assessment of the two fungal stem pathogens has revealed that the symptoms of *B. dothidea* and *T. zuluensis* can be present separately or concurrently at varying levels on trees in the population in the form of a fungal stem disease complex.

In general, tree breeding strategies use pedigree information to estimate genetic merit, often in trials with large numbers of individuals in open-pollinated families. The availability of a reference genome sequence of *E. grandis* (Myburg et al., 2014) and the development of a robust single-nucleotide polymorphism (SNP, EUChip60K) chip for high-throughput genotyping in multiple eucalypt species (Silva-Junior et al., 2015) have created opportunities for implementing new breeding strategies based on the genomic prediction of breeding values. While conventional pedigree relationships represent the average proportion of shared alleles, SNP markers can track Mendelian segregation patterns enabling the detection of unknown (cryptic) relationships and more precise estimation of known relationships (Habier et al., 2007; Hayes et al., 2009; Hill and Weir, 2010). However, the genotyping of all individuals in large open-pollinated tree breeding populations would be prohibitively expensive. Single-step genomic (ssG)BLUP analysis is an attractive alternative that blends the known pedigree of the entire population with the genomic relationship matrix of a subset of genotyped individuals (Legarra et al., 2009; Misztal et al., 2009; Aguilar et al., 2010; Christensen and Lund, 2010). Thereby, ssGBLUP analysis extends the benefits of applying of genomic selection to non-genotyped individuals (Legarra et al., 2014), therefore allowing for multivariate and univariate analysis (Guo et al., 2014) in livestock (Lourenco et al., 2015; Ma et al., 2015) and forest trees (Ratcliffe et al., 2017; Klapste et al., 2018, 2020; Cappa et al., 2019).

Improving forest plantation productivity requires recurrent selection of multiple traits, such as growth, wood quality, and tolerance to pests and pathogens. A multivariate analysis involves estimating genetic correlations between traits to understand their correlated responses (Burdon, 1977). The correlated phenotypes of growth and pest and disease traits are attributable to shared genetic factors (pleiotropy) and/or linked genetic factors (linkage disequilibrium) and their interrelationships with environment factors (Falconer and Mackay, 1996). Being able to partition these components will help improve breeding strategies for correlated traits (Chen and Lubberstedt, 2010).

In this study, we measured breeding trials of *E. grandis* composed of trees from three half-sib pedigree linked generations and some unrelated families for diameter growth at breast height, tolerance to stem disease caused by the co-occurrence of *B. dothidea* and *T. zuluensis* (*BotryoTera*), and tolerance to leaf gall caused by *L. invasa* (*Lepto*). The study aimed to obtain genetic parameters and genetic gains for growth, pest, and pathogen tolerance in this multi-generation breeding trial comparing ABLUP (pedigree-based BLUP analysis) and ssGBLUP models. We further investigated the additive genetic correlations and genotype-by-environmental (G × E) interactions of diameter growth and tolerance to *Lepto* and *BotryoTera*. Based on the results, we discuss the utility of genomic selection in *E. grandis* for simultaneous improvement of growth and tolerance to the gall wasp and fungal stem disease.

## Materials and Methods

### Breeding History and Phenotyping of the Study Population

**FIGURE 1:**
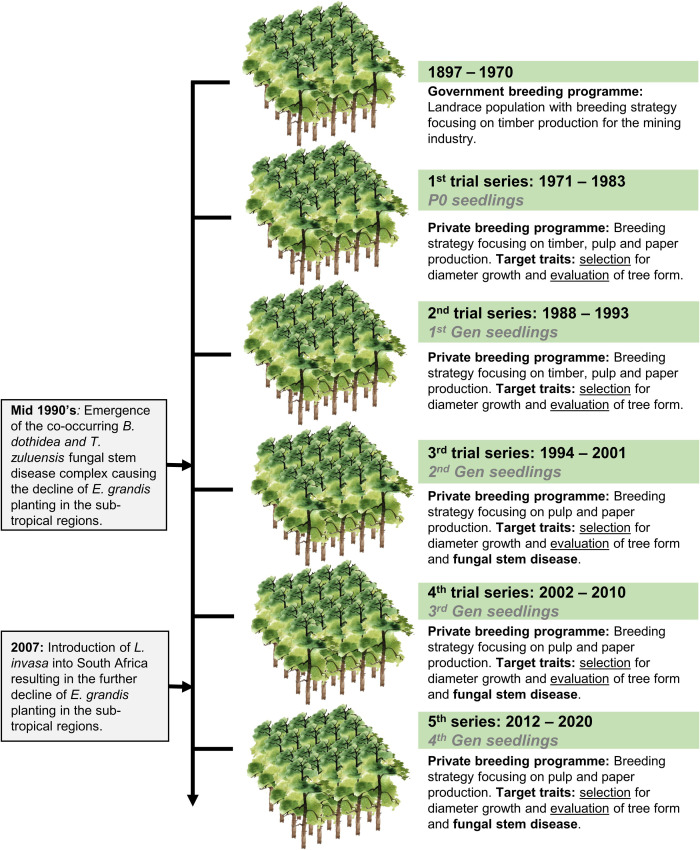
Historical overview of *E. grandis* breeding in South Africa, including a transition from government to private breeding and introduction of major pest and pathogens. The trial series timeline, as well as the generational timeline, are shown. Selection strategies are noted for each trial series, shifting from timber to pulp and paper related traits, as well as pest and disease tolerance. Selection refers to the selection of phenotyped individuals based on their breeding values, whereas evaluation refers to the selection of individuals based on visual screening without breeding values.

**FIGURE 2:**
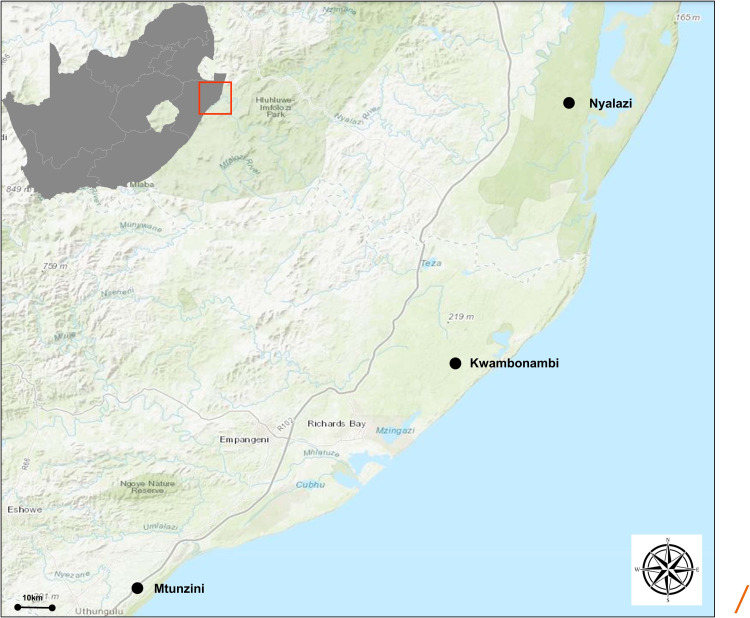
Geographical representation of the trial sites in the KwaZulu Natal province, South Africa. The region has a sub-tropical climate. The distance (straight line) between Mtunzini and Nyalazi is 112 km. The details of the environmental conditions are in Supplementary Table 1. Darker shades of green indicate nature reserves.

**FIGURE 3:**
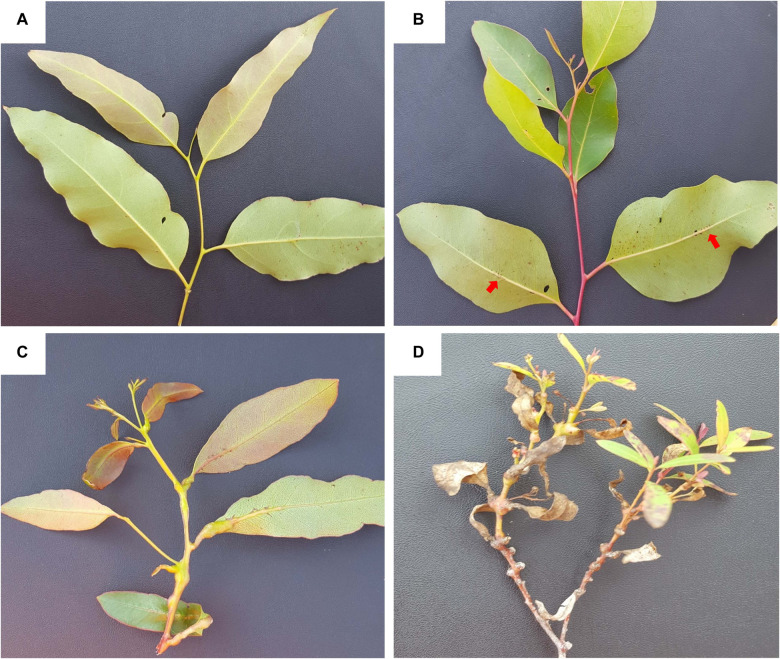
Symptoms and incidence scores of *Leptocybe invasa* (*Lepto*). **(A)** Score 4 – No evidence of an attack on the leaf midrib or petiole, **(B)** Score 3 – Evidence of attack on the leaf midrib or petiole without galls (indicated by red arrows), **(C)** Score 2 – Evidence of attack on the leaf midrib or petiole with galls, and **(D)** Score 1 – Evidence of a lethal outcome of an attack on the leaf midrib or petiole with galls.

**FIGURE 4:**
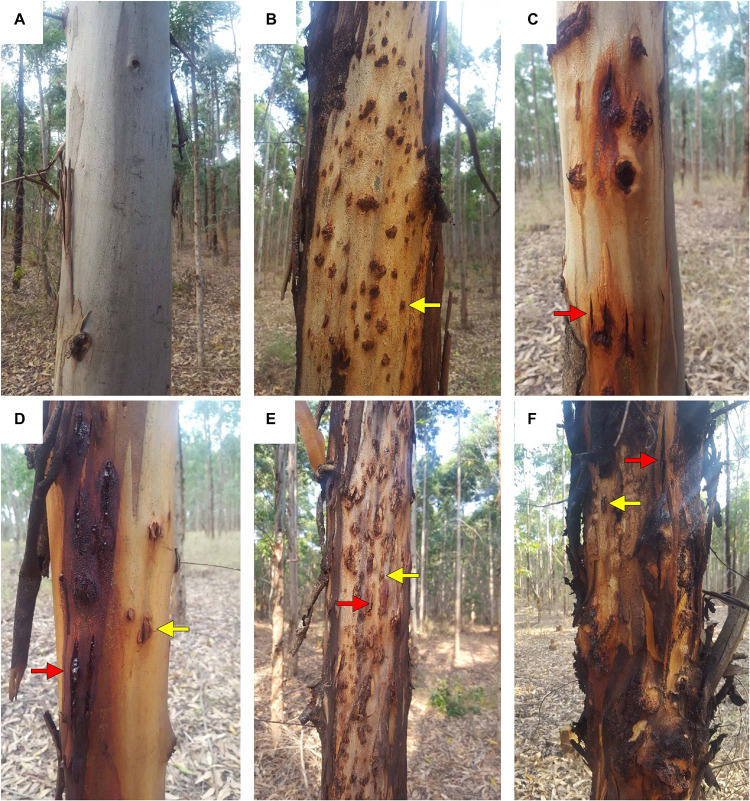
Symptoms and incidence scores for *Botryosphaeria*/*Teratosphaeria* stem disease complex (*BotryoTera*). **(A)** A score of 6 represents trees with no spots/cracks or redness. **(B)** A score of 5 represents trees with *T. zuluensis* spots with redness. **(C)** A score of 4 is given for trees with *B. dothidea* cracks with redness. **(D)** A score of 3 shows a tree with *T. zuluensis* spots and *B. dothidea* cracks with redness. **(E)** A score of 2 represents trees with heavy *T. zuluensis* spots and *B. dothidea* cracks with redness. **(F)** A score of 1 represents trees with heavy *T. zuluensis* spots and *B. dothidea* cracks with redness and cankers.

*Eucalyptus grandis* W. Hill ex Maiden was introduced to South Africa in the early 1900s and included various government breeding populations as a timber resource for the mining industry. Private breeding programs only started in the early 1970s, initiated from government landrace breeding populations. Breeding objectives for these landrace breeding populations gradually shifted to target traits for pulp and paper products rather than timber production in successive generations and trial series (Figure 1). We had access to seed from two first-generation selections from the 2nd trial series in this study population, with 32 selections from the 3rd trial series as our third-generation families and 28 selections from the 4th trial series as our fourth-generation families (Supplementary Table 1). Also included in the study was 33 unrelated (no pedigree link) families as controls, with seed sourced in the early 1990s from selections in Swaziland. The 93 half-sib pedigree linked families and the 33 unrelated control families were planted across three sites Mtunzini, Kwambonambi, and Nyalazi in KwaZulu Natal, a sub-tropical region in South Africa (Figure 2 and Supplementary Table 1). Families from the different generations were planted together in the three trial sites. The experimental design was a randomized complete block planted at single tree plots at 15 replicates per family. Field tolerance to *Lepto* was assessed at age 1 using a four-scale incidence score in which trees with a score of 4 shows no evidence of an attack on the leaf midrib or petiole, a score of 3 shows evidence of an attack on the leaf midrib or petiole without galls, and a score of 2 indicates trees with an attack on the leaf midrib or petiole with galls. Trees with a score of 1 present a lethal outcome from an attack on the leaf midrib or petiole with galls (Figure 3). Field tolerance to *BotryoTera* was assessed at age 3 using an incidence score in which a score of 6 represents trees with no spots/cracks or redness and trees with a score 5 show symptoms of *T. zuluensis* spots with redness, whereas trees with a score of 4 have *B. dothidea* cracks with redness. Trees with a score of 3 shows symptoms with *T. zuluensis* spots and *B. dothidea* cracks with redness, and a score of 2 represents trees with heavy *T. zuluensis* spots, and *B. dothidea* cracks with redness, and a score of 1 represents trees with heavy *T. zuluensis* spots and *B. dothidea* cracks with redness and cankers (Figure 4). Diameter growth at breast height (1.3 m over-bark) was measured at age 4.

### Genotyping of the Study Population

DNA was extracted from leaves using the NucleoSpin DNA extraction kit (Machery-Nagel, Germany). The *Eucalyptus* (EUChip60K) SNP chip as described by Silva-Junior (Silva-Junior et al., 2015) available from GeneSeek (Neogen, Lansing, MI, United States) was used to genotype 964 trees across the families and trials (Supplementary Table 1). Of the 95 families in the trials, 93 contained a subset of 964 genotyped trees ranging from 2 to 24 trees per family. The two second-generation families were not genotyped. An average of four trees per family were genotyped of the unrelated families. For the third generation, 15 trees per family were genotyped, while in the fourth generation, 14 per family were genotyped. Of the 64,639 markers on the SNP chip (Silva-Junior et al., 2015), there are a total of 14,347 informative SNP markers with GenTrain score ranging from 0.37 to 0.93. Retained markers had a call rate of above 90% and a minor allele frequency above 0.05. The SNP genotypes frequencies of the 14,347 markers were AA (0.307), GG (0.283), AG (0.270), CC (0.068), AC (0.065), and 0.007 missing. The number of SNP markers distributed on linkage groups ranged from 1018 (Chromosome 1) to 1877 (Chromosome 10). The SNP marker frequencies and distribution analysis were performed with the *synbreed* 0.10-2 R package (Wimmer et al., 2012) and the imputing of the missing SNP data based on allelic distribution, assuming Hardy–Weinberg equilibrium.

### Statistical Analyses

#### Mixed Model Analysis

Linear mixed models were fit to estimate variance components and solve mixed model equations to obtain solutions for fixed and random effects. The matrix notation for the linear mixed models used is as follows:

(1) y=Xβ+Zu+ε

where *y* is a vector of phenotypes, *X* is the design matrix for the fixed effects (site), β is the vector of the fixed effect coefficients (intercept site), *Z* is an incidence matrix for the random effects of individual trees, *u* is the vector of random effect coefficients (genotype, genotype by site interaction, replication effect nested in site effect), and ε is the vector of residual effect coefficients. The expectations of *y*, *u*, and *e* are *E*(*y*) = *X*β, $E\left(\mu \right)=N\left(0,\sigma_{u}^{2}\right),$ and *E*(ε) = 0 and the variances are Var(y)=V=ZGZ+′R, $Var\left(\varepsilon \right)=R=N\left(0,I\sigma_{e}^{2}\right)$, and $Var\left(u\right)=\text{G}=A\sigma_{u}^{2}$, respectively, where **A** is the relationship matrix of the random effects, $\sigma_{\varepsilon }^{2}$ is the variance associated with the residuals, and $\sigma_{u}^{2}$ is the variance associated with the random effects. The assumptions of residual matrix *R* was relaxed to have a heterogeneous error variance across the environments. Similarly, the assumption of the *G* matrix was relaxed to model full G × E and heterogeneous genetic variances at each site (*s* + 1 variance parameters), where *s* is the number of environments (Isik et al., 2017). Empirical breeding value prediction for the half-sibs was performed by solving the mixed model equations.

(2) [XX′XZ′ZX′ZZ′+A-1λ][βu]=[Xy′Zy′]

where *A*^−1^ is the inverted additive genetic relationship matrix derived from the pedigree and $\lambda =\frac{\sigma_{e}^{2}}{\sigma_{u}^{2}}$ is the shrinkage factor. The genomic relationship matrix **G** from the genotyped trees was computed as described in VanRaden (2008):

(3) $$\text{G}=\frac{\left(\text{Z}-\text{P}\right)\left(Z-P\right){}^{\prime }}{2\sum pi\left(1-p_{i}\right)}$$

where *Z* and *P* are two matrices of dimension *n* (individuals) × *p* (markers). The base pair calls were transformed into gene content values of the minor alleles at each SNP loci in each individual in matrix *Z*, with elements −1 (homozygote major allele), 0 (heterozygote), and 1 (homozygote minor allele). The frequencies of the genotypes were 0.584, 0.338, and 0.078, respectively. The allele frequencies in matrix *P* are presented as 2(*p*_*i*_−0.5), where *p*_*i*_ is the observed allele frequency at the marker *i* for all individuals. The 2∑*pi*(1−*p*_*i*_) is the variance of alleles summed across all the loci. A ssGBLUP model was fitted using a blended relationship (*H*) matrix, incorporating the (*G*) matrix of genotyped trees that are linked to the non-genotyped trees by the half-sib pedigree (*A*) matrix (Legarra et al., 2009; Aguilar et al., 2010; Christensen and Lund, 2010).

The *H* matrix used in the ssGBLUP was formulated as follows: where *u* is a vector of genetic effects with variances $Var\left(u\right)=A\sigma_{u}^{2}$. Within the genetic effects (*u*), there are non-genotyped and (*u*_1_) and genotyped (*u*_2_) individuals partitioned in the *A* matrix as:

(4) $$\text{A}=\left[\begin{array}{cc} {\text{A}}_{11} & A_{12}\\ A_{21} & A_{22} \end{array}\right]$$

where *A*_11_ is the relationship matrix of non-genotyped individuals, *A*_22_ is the relationship matrix for the genotyped individuals, and *A*_12_ and its transpose *A*_21_ are the covariances between the genotyped non-genotyped individuals. We then replaced the *u*_2_ genetic effects with the pedigree relationship of *A*_22_ with their *G* matrix as constructed in Eq. 3. The relationship between the non-genotyped and (*u*_1_) and genotyped (*u*_2_) individuals in *A*_12_ and *A*_21_ is then adjusted by the *G* matrix via the pedigree relationship of all other individuals in the *H* matrix (Legarra et al., 2009):

(5) $$\text{H}=\left[\begin{array}{cc} A_{11}+A_{12}A_{22}^{-1}\left(G-A_{22}\right)A_{22}^{-1}A_{21} & A_{12}A_{22}^{-1}G\\ GA_{22}^{-1}A_{21} & G \end{array}\right]$$

The upper left corner of the *H* matrix is the variance of the *u*_1_ individuals, with $Var\left(u_{1}\right)=\left[A_{11}A_{12}A_{22}^{-1}\left(G-A_{22}\right)A_{22}^{-1}A_{21}\right]\sigma_{A}^{2}$, and $Var\left(u_{2}\right)=G\sigma_{A}^{2}$ and $Cov\left(u_{1},u_{2}\right)=A_{12}A_{22}^{-1}G\sigma_{A}^{2}$. The inverse of the *H* matrix is:

(6) $$H^{-1}=A^{-1}+\left[\begin{array}{cc} 0 & 0\\ 0 & G^{-1}-A_{22}^{-1} \end{array}\right]$$

Variance components from the ABLUP and ssGBLUP were estimated along with the heritability for diameter growth and *Lepto* and *BotryoTera* tolerance across and within the three sites.

#### Multivariate Analysis

A multivariate linear mixed model was fitted to estimate additive genetic correlations between three pairs of traits as described in Isik et al. (2017), following the multivariate model general design:

(7) $$y_{n\times d}=X_{n\times \left(p+1\right)}\beta_{\left(p+1\right)\times d}+Z_{n\times r}u_{r\times d}+\varepsilon_{n\times d}$$

where *n* is the number of rows of individuals and *d* is the number of dependent variables (traits). The design matrix *X* has the dimensions *n* = (*p*1), where *p* is the number of fixed estimators, which are replication nested in location for the traits, and the additional column is added for the intercept. β is the matrix of coefficients of fixed predictor effects to be estimated with dimensions (*p*1) = *d*. The rows of β correspond to predictor variables, and the columns are response variables. The design matrix of *Z* has dimensions *n* = *r*, where *r* is the number of random effects (individual trees) per trait, and *u* is a *r* = *d* matrix of the random effects.

The *G* and *R* variance–covariance matrices of the multivariate model were designed with the variances for the three traits on the diagonal and the covariances between the traits on the off-diagonals:

(8) $$G=A⊗\left[\begin{array}{ccc} \sigma_{A11}^{2} & \sigma_{A12} & \sigma_{A13}\\ \sigma_{A21} & \sigma_{A22}^{2} & \sigma_{A23}\\ \sigma_{A31} & \sigma_{A32} & \sigma_{A33}^{2} \end{array}\right]$$

(9) $$R=I_{m}⊗\left[\begin{array}{ccc} \sigma_{\varepsilon 11}^{2} & \sigma_{\varepsilon 12} & \sigma_{\varepsilon 13}\\ \sigma_{\varepsilon 21} & \sigma_{\varepsilon 22}^{2} & \sigma_{\varepsilon 23}\\ \sigma_{\varepsilon 31} & \sigma_{\varepsilon 32} & \sigma_{\varepsilon 33}^{2} \end{array}\right]$$

where the *G* matrix is the direct product of the *A* matrix (pedigree relationship) for the ABLUP model and substituted with the *H* matrix for the ssGBLUP model with an unstructured, heterogeneous variance and covariance structure, where each environment has a unique genetic variance, and each pair of the environments has a unique covariance, with an *s*(*s*1)2 variance parameter (Isik et al., 2017). The *R* matrix is the direct product of the identity matrix (*I*_*m*_) with *m* dimensions, *m* is the number of genotypes with variance $\sigma_{\varepsilon 1}^{2}$for diameter growth, $\sigma_{\varepsilon 2}^{2}$ for *BotryoTera*, and $\sigma_{\varepsilon 3}^{2}$ for *Lepto* and their covariances nested within.

The construction of the expected additive (*A* matrix) and the realized genomic (*G*) was calculated using the package *synbreed* 0.10-2 (Wimmer et al., 2012) in the R environment v3.5.3. The blended genetic relationships and its inverse were obtained using scripts according to Isik et al. (2017). All the statistical models were performed using ASReml software v4.1 (Gilmour et al., 2015).

#### Expected Direct and Indirect Genetic Gains

The direct genetic gains for *diameter* growth and *Lepto* and *BotryoTera* tolerance were calculated from the ABLUP and ssGBLUP models breeding value predictions. The selection differential was based on the top 10% of individuals for direct selection. The indirect responses of the remaining traits were calculated based on the ranking of the direct selections. The percentage expected genetic gains were calculated as the fraction of the selection differential over the population mean.

## Results

### Genetic Parameters

To assess the increased accuracy of the ssGBLUP model, we compared the heritability estimates from ssGBLUP with those from ABLUP analysis. The ssGBLUP model generally produced lower heritability estimates compared to the ABLUP model for the three sites (Table 1). The exception was the heritability estimates for *BotryoTera* tolerance in Kwambonambi and Nyalazi, which were higher for ssGBLUP (0.45 vs. 0.29 and 0.11 vs. 0.08, respectively). Overall, the Kwambonambi site produced the highest heritability values ranging from 0.29 to 0.63 (ABLUP) and from 0.45 to 0.70 (ssGBLUP) across the traits (Table 1). In contrast, the heritability estimates for *Lepto* tolerance from the ABLUP and ssGBLUP models were the highest at 0.71 and second highest at 0.38, respectively, in Nyalazi, while the estimates for diameter growth and *BotryoTera* tolerance at the Nyalazi site were reasonably low, ranging from 0.07 to 0.11 for the ABLUP and ssGBLUP models, respectively (Table 1). The overall heritability estimates across sites were higher for the ABLUP model with *Lepto* tolerance moderately high at 0.54, diameter growth at 0.33, and *BotryoTera* tolerance at 0.23 (Table 2). The heritability estimates with the ssGBLUP across sites were lower with *Lepto* tolerance at 0.36, diameter growth at 0.25, and *BotryoTera* tolerance at 0.23 (Table 2). The heritability estimates for ssGBLUP may be more accurate due to the blended pedigree relationship matrix increased precision.

**TABLE 1:** Site-specific variance components and genetic parameters estimated using the ABLUP and ssGBLUP mixed models for diameter growth, *BotryoTera* and *Lepto* tolerance.

	$\sigma_{u}^{2}\left(se\right)$	$\sigma_{e}^{2}\left(se\right)$	*h*^2^(*se*)
**Diameter**			
**ABLUP**			
Mtunzini	6.655 (0.281)	2.360 (0.655)	0.36 (0.092)
Kwambonambi	13.193 (0.662)	8.250 (1.945)	0.64 (0.129)
Nyalazi	11.928 (0.547)	0.884 (0.579)	0.07 (0.048)
**ssGBLUP**			
Mtunzini	6.670 (0.277)	1.620 (0.504)	0.24 (0.072)
Kwambonambi	13.592 (0.682)	7.852 (1.487)	0.58 (0.092)
Nyalazi	11.958 (0.552)	0.779 (0.582)	0.07 (0.048)
***BotryoTera***			
**ABLUP**			
Mtunzini	1.450 (0.055)	0.424 (0.115)	0.29 (0.0752)
Kwambonambi	2.334 (0.099)	0.115 (0.203)	0.30 (0.0823)
Nyalazi	1.411 (0.059)	0.109 (0.056)	0.08 (0.0393)
**ssGBLUP**			
Mtunzini	1.447 (0.053)	0.222 (0.077)	0.15 (0.052)
Kwambonambi	2.404 (0.110)	1.088 (0.227)	0.45 (0.083)
Nyalazi	1.418 (0.060)	0.154 (0.073)	0.11 (0.051)
***Lepto***			
**ABLUP**			
Mtunzini	0.454 (0.017)	0.161 (0.039)	0.36 (0.080)
Kwambonambi	0.762 (0.035)	0.524 (0.105)	0.70 (0.118)
Nyalazi	0.764 (0.037)	0.542 (0.112)	0.71 (0.125)
**ssGBLUP**			
Mtunzini	0.452 (0.016)	0.110 (0.026)	0.24 (0.055)
Kwambonambi	0.770 (0.033)	0.538 (0.070)	0.70 (0.072)
Nyalazi	0.744 (0.031)	0.281 (0.049)	0.38 (0.059)

*The residual variance $\left(\sigma_{e}^{2}\right)$, additive genetic variance $\left(\sigma_{u}^{2}\right)$, narrow-sense heritability (h^2^), and their standard errors (se) are shown.*

**TABLE 2:** Overall variance components and genetic parameters across the three sites for solving ABLUP and ssGBLUP mixed models for diameter growth, *BotryoTera*, and *Lepto* tolerance.

	$\sigma_{u}^{2}\left(se\right)$	$\sigma_{e}^{2}\left(se\right)$	*h*^2^(*se*)
**ABLUP**			
Diameter	10.581 (0.314)	3.450 (0.720)	0.33 (0.063)
*BotryoTera*	1.732 (0.044)	0.407 (0.092)	0.24 (0.051)
*Lepto*	0.659 (0.021)	0.357 (0.059)	0.54 (0.077)
**ssGBLUP**			
Diameter	10.729 (0.313)	2.733 (0.469)	0.26 (0.040)
*BotryoTera*	1.755 (0.046)	0.396 (0.071)	0.23 (0.038)
*Lepto*	0.655 (0.017)	0.238 (0.024)	0.36 (0.032)

*The residual variance$\left(\sigma_{e}^{2}\right)$, additive genetic variance$\left(\sigma_{u}^{2}\right)$, narrow-sense heritability (h^2^), and its standard error (se) are presented.*

### ssGBLUP Additive and Type-B Genetic Correlations

The additive genetic correlations of diameter growth and *Lepto* tolerance estimated with the ssGBLUP model was high at 0.78 (Table 3, Eq. 7). In contrast, the additive genetic correlation of diameter growth and *BotryoTera* tolerance was moderate at −0.38. The additive genetic correlation for *BotryoTera* and *Lepto* tolerance was also moderate at −0.47 (Table 3). These results suggest that tandem improvement of diameter growth and *Lepto* tolerance is possible, but they predict a negative response in *BotryoTera* tolerance, which presents a challenge to breeders. The overall Type-B genetic correlation (Eq. 7) was high, ranging from 0.77 to 0.81 for the three traits associated with small standard errors (Table 4), suggesting low G × E interactions across the sites.

**TABLE 3:** Additive genetic correlations (*r*_*g*_) of diameter growth, *BotryoTera*, and *Lepto* tolerance based on ABLUP and ssGBLUP models with standard errors in the parenthesis.

	*BotryoTera*	*Lepto*
**ABLUP**		
Diameter	−0.46(0.116)	0.81 (0.054)
*BotryoTera*		−0.47(0.111)
**ssGBLUP**		
Diameter	−0.38(0.106)	0.78 (0.055)
*BotryoTera*		−0.47(0.089)

**TABLE 4:** Overall Type-B genetic correlation (*r*_*B*_) across sites for diameter growth, *BotryoTera*, and *Lepto* tolerance based on ABLUP and ssGBLUP models with standard errors in the parenthesis.

	*r*_*B*_(*se*)
**ABLUP**	
Diameter	0.90 (0.096)
*BotryoTera*	0.99 (0.000)
*Lepto*	0.88 (0.055)
**ssGBLUP**	
Diameter	0.80 (0.140)
*BotryoTera*	0.81 (0.147)
*Lepto*	0.77 (0.072)

### Trait Performance Across Site and Generations

**FIGURE 5:**
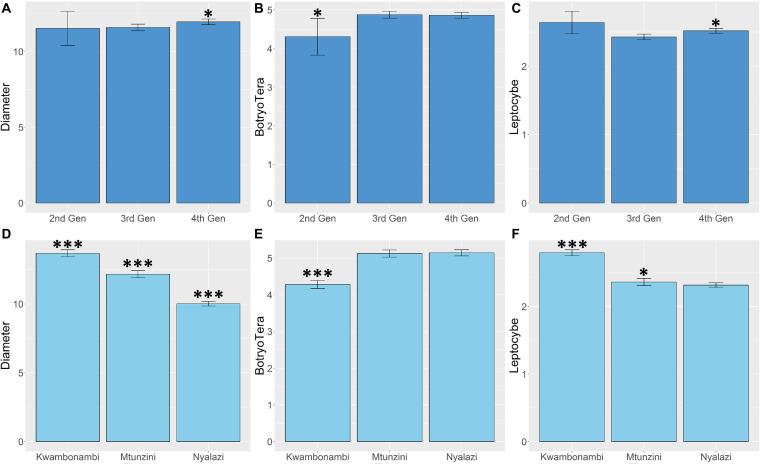
Marginal trait means with error bars indicating the 95% confidence interval. **(A)** Mean diameter growth (cm) for families in the three sites. **(B)** The mean *Lepto* tolerance score for families in the three sites. **(C)** The mean *BotryoTera* tolerance score for families in the three sites. **(D)** Mean diameter growth (cm) for families in the three generations. **(E)** The mean *Lepto* tolerance score for families in the three generations. **(F)** The mean *BotryoTera* tolerance score for families in the three generations. Student *t-*test was performed to assess the significant difference between the means, *p* < 0.05 (*) and *p* < 0.001 (***).

Diameter growth and the *Lepto* incidence scores resembled a normal distribution (Supplementary Figure 1). *BotryoTera* incidence scores had a high frequency of score 6, representing uninfected stems, and Kwambonambi has a high frequency of score 3 (Supplementary Figure 1). The latter may be ascribed to the second-generation families’ higher susceptibility (Figure 5B and Supplementary Figure 2). The Kwambonambi site had the lowest mean *BotryoTera* tolerance compared to the Nyalazi and Mtunzini (Figure 5E). The average diameter growth improved by 3.2% from the third to the fourth generation (Figure 5A), whereas *Lepto* tolerance improved by 3.6% (Figure 5C). The improvement in diameter growth is driven by recurrent selection over the generations with *Lepto* tolerance benefiting from its strong additive genetic correlation with diameter growth (Table 3). There was a 13.3% improvement of *BotryoTera* tolerance from the second to the third generation; however, it was unchanged from the third to the fourth generation (Figure 5B). The apparent absence in genetic gain for *BotryoTera* tolerance from the third to the fourth generation is in part due to the moderately negative genetic correlation with diameter growth (Table 3). The above results suggest that a revised breeding strategy is needed to improve the three traits simultaneously.

### Correlated Response Based on ssGBLUP Breeding Values

The direct genetic gains estimated for diameter growth and *Lepto* tolerance were 12.4% and 24.7%, respectively, with *BotryoTera* at 9.8% (Table 5). There is an indirect loss of 3.4% in *BotryoTera* tolerance and a gain of 10.0% in *Lepto* tolerance when selecting for diameter growth. Direct selection for *BotryoTera* tolerance would result in an expected indirect loss of 5.6% for diameter growth and 6.5% for *Lepto* tolerance. However, direct selection of *Lepto* tolerance would result in an expected gain of 6.0% for diameter growth and loss of 3.8% in *BotryoTera* tolerance (Table 5). Together, these results illustrate the challenge of achieving genetic gains for all three of these traits and the need for customized breeding strategies to deal with this challenge.

**TABLE 5:** Expected genetic gains (%) based on the top 10% selected individuals in the population for diameter growth, *BotryoTera*, *Lepto* tolerance, and the indirect response in the expected genetic gains of the paired traits.

		Diameter	*BotryoTera*	*Lepto*
**ABLUP**				
Direct response		15.1	8.9	32.2
Indirect response	Diameter	**15.1**	–3.5	9.9
	*BotryoTera*	–6.1	**9.3**	–8.0
	*Lepto*	5.9	–1.9	**26.4**
**ssGBLUP**				
Direct response		12.4	9.8	24.7
Indirect response	Diameter	**12.4**	–3.4	10.0
	*BotryoTera*	–5.6	**10.1**	–6.5
	*Lepto*	6.0	–3.8	**20.9**

*The bold diagonals are the direct response with the off-diagonal as the indirect responses.*

## Discussion

Pest and pathogens are significant risk factors in forest plantations (Wingfield et al., 2015). These risk factors are highlighted in African agroforestry systems affecting indigenous and natural forests (Graziosi et al., 2020). Mitigation of these risk factors will require recognizing the parallels and synergies in management methods between pest and pathogen studies (Jactel et al., 2020) and integrating system genetic and systems biology (Naidoo et al., 2019) particularly in this genomic era (Naidoo et al., 2014). The continued improvement of economic traits such as volume growth, density, and pulp yield in the context of pest and pathogen challenges is vital. Here, we combined phenotypic data for a large half-sib breeding trial with genotypic data for a subset of siblings in a single-step genomic BLUP approach to estimate genetic parameters and response to selection for diameter growth and *BotryoTera* and *Lepto* tolerance in *E. grandis* breeding population. We also proposed a practical genomic selection breeding strategy that is likely to improve all three traits in *E. grandis.* One of the study strengths was the availability of replicated trials with *BotryoTera* infections and *Lepto* infestation across all three sites.

Furthermore, the study benefited from planting pedigree-linked families from three successive generations in the same space and time. Therefore, the trials provided an opportunity to evaluate the outcomes of three different artificial selection regimes applied in successive generations. A limitation was the inability to score *B. dothidea* and *T. zuluensis* infections separately, which we mitigated by developing a combined phenotypic score (Figure 3). Diameter growth and *BotryoTera* and *Lepto* tolerance had moderate heritability estimates (0.25–0.36, Table 2). Diameter growth and *Lepto* tolerance had a strong positive additive genetic correlation. However, both were negatively correlated with *BotryoTera* tolerance, though the correlations were not strong. This presents a challenge to achieve genetic gains in all three traits simultaneously.

### Genetic Parameters for Diameter Growth and *Lepto* and *BotryoTera* Tolerance

Coefficients of relationship from pedigree data are expectations and do not represent the actual genome shared between relatives, estimated from various allelic frequency parameters (Forni et al., 2011). Forest trees with deep full-sib pedigrees have estimated coefficients of relationship that are much closer to the actual genetic relationships (Batholome et al., 2016; Chen et al., 2018). However, more precise coefficients of relationship are estimated using DNA markers such as SNPs (Habier et al., 2007; Hayes et al., 2009). When expected genetic relationships are combined with the genome estimated relationships, this precision can be extrapolated to the *A* matrix with the blended *H* matrix used in ssGBLUP analyses (Legarra et al., 2009; Aguilar et al., 2010). Half-sib pedigree relationships do not include cryptic genetic relationships in the population, in some instances leading to biased estimation of additive genetic variances (Ratcliffe et al., 2017).

In this study, we generally observed lower heritability estimates from ssGBLUP compared to ABLUP (Table 2). Lower additive genetic correlation estimates were also observed for ssGBLUP compared to ABLUP (Table 3). Luo et al. (2014) presented heritability estimates of *Lepto* tolerance in *E. camaldulensis* and *E. tereticornis* breeding populations in China of 0.54 and 0.52, respectively. da Silva et al. (2020), also presented heritability estimated from multiple *Eucalyptus* species ranging from 0.27 to 0.68, with *E. grandis* at 0.58. These heritability estimates are similar to what we obtained in our study at 0.54 for *E. grandis* (Table 2). The *Lepto* tolerance scores in the study by Luo et al. (2014) were based on the proportion of the canopy affected, with a score of 0 indicating no symptoms on the canopy and a score of 4 meaning greater than 75% of the canopy affected (Thu et al., 2009).

In contrast, our scoring system was not based on canopy proportions, but rather the severity of gall formation with a score of 4 indicating no evidence of gall formation and a score of 1 indicating lethal outcome from gall formation in both mid-ribs and petioles of the leaves (Figure 3). Luo et al. (2014) reported a moderately negative genetic correlation between tree height (at 9 months) and *Lepto* susceptibility in *E. camaldulensis* at −0.33 and for *E. tereticornis* at −0.47. Due to the inverted scores used in our study, we report a positive genetic correlation (0.78) with diameter growth at 48 months (Table 3). These results suggest that vigorous tree growth is positively related to tolerance to *L. invasa*. Plant growth regulators are well-characterized phytohormones involved in influencing plant development and abiotic stress responses (Wani et al., 2016) and pest tolerance (Harun-or-Rashid and Chung, 2017). There is evidence to suggest that the microbiome of the maternal environment may affect the performance of their progeny and tolerance to pathogens in *E. grandis* (Vivas et al., 2017). A study to characterize the relationship of maternal and/or progeny microbiomes, phytohormones, and their interactions, on superior tree growth and health, is warranted.

### Genotype-by-Environment Interaction and Trait Performance

The mean annual precipitation of the three sites in the subtropical region of South Africa decreases from South to North, tracking the increase in the mean annual temperature maximum (Figure 2). Therefore, Nyalazi in the North is on average warmer and drier compared to Mtunzini in the South, which is on average colder and wetter, whereas Kwambonambi has mid-ranged environmental conditions (Supplementary Table 1). The pairwise Type-B genetic correlation for diameter growth and *Lepto* and *BotryoTera* tolerance across the sites ranged from 0.77 to 0.81 (Table 4), indicating low G × E interaction. The Nyalazi trial was surrounded by a commercial stand of *E. grandis* × *E. camaldulensis* (G × C) clone that was highly susceptible to *L. invasa*. The G × C hybrid genotype has been shown in the literature to be susceptible to *L. invasa* (Thu et al., 2009; Luo et al., 2014). The G × C clone planted in the Nyalazi site had an increased infestation of *L. invasa* translating into the high frequency of *Lepto* tolerance score 2 in the trial and much lower frequency of *Lepto* tolerance score 3 and 4 (Supplementary Figure 1). In Mtunzini, there was also an increased frequency of *Lepto* score 2; however, the trial was surrounded by a tolerant *E. grandis* × *E. urophylla* (G × U) clone (Supplementary Figure 1). There are above-average actively growing shoots in Mtunzini due to its favorable environmental conditions (Supplementary Table 1). These actively growing shoots are targets for *L. invasa* infestation. The heritability estimates of *Lepto* tolerance in Mtunzini and Nyalazi were adjusted lower from 0.35 to 0.24 and 0.71 to 0.38, respectively, by the ssGBLUP model (Table 1). It is not clear why the heritability correction in Nyalazi was so significant compared to that in Mtunzini.

In Kwambonambi, the mid-range environmental conditions to Mtunzini and Nyalazi, which was also surrounded by a tolerant G × U clone, *Lepto* tolerance showed similar heritability estimates between ABLUP (0.69) and ssGBLUP (0.70) and for diameter growth ABLUP (0.63) and ssGBLUP (0.58) (Table 1). The similar heritability estimates in Kwambonambi of diameter growth and *Lepto* tolerance may result from their relatively high positive additive genetic correlation. The estimated marginal means for diameter growth and *Lepto* tolerance in Kwambonambi further support this relationship (Figures 5D,F).

There is an increased incidence of *BotryoTera* tolerance score 3 in Kwambonambi (Supplementary Figure 1), resulting from the increased susceptibility from the second-generation families (Supplementary Figure 2). *BotryoTera* appeared as a fungal stem disease in the mid- to late 1990s, which means that the first-generation parents (second-generation families) were selected in the absence of the *BotryoTera* disease explaining the higher susceptibility of the second generation families. The environmental conditions at the Kwambonambi site are optimal for diameter growth, and, due to the negative correlation with *BotryoTera* tolerance, there was high susceptibility to *BotryoTera* in Kwambonambi (Figure 5E). Diameter growth and *Lepto* and *BotryoTera* tolerance in the Kwambonambi site, which is the mid-range of Nyalazi and Mtunzini environmental conditions, seem to reflect the trait performances, corresponding to their additive genetic correlation.

### Generational Performance for Diameter Growth and *Lepto* and *BotryoTera* Tolerance

Recurrent selection in tree breeding ensures the gradual improvement of target economic traits over generations. Such efforts are under threat from pest and pathogen pressures as well as climate change (Wingfield et al., 2015). Reversing the decline of *E. grandis* in the subtropical region of South Africa due to *L. invasa* gall wasp and the co-occurrence of *B. dothidea* and *T. zuluensis* fungal stem disease is vital. *BotryoTera* fungal stem disease was discovered and described in South Africa in the early to mid-1990s (Smith et al., 1994; Wingfield et al., 1996). This meant that selections or evaluations in the government landrace breeding populations did not involve *BotryoTera* tolerance until the first generation in the 2nd trial series and onwards in the private breeding population (Figure 1), evidenced by the high *BotryoTera* incidence score 3 (Supplementary Figure 2) of the second-generation families in particular in the Kwambonambi site (Supplementary Figure 1). Evaluation for *BotryoTera* tolerance in the second generation resulted in the increased tolerance in the third generation and maintained in the fourth generation (Figure 5B). When looking at the high frequency of *BotryoTera* score 6 in Supplementary Figures 1, 2, it does suggest that the evaluation strategy has had a limited role to play in improving *BotryoTera* tolerance, because this trait seems to have plateaued in the last generations. The limitation of the evaluation strategy for *BotryoTera* tolerance is that selection was only performed within families already selected for diameter growth and further compounded by the fact that *BotryoTera* tolerance is negatively correlated with diameter growth.

*Leptocybe invasa* was reported in South Africa in 2007 (Neser et al., 2007), coinciding with the third generation tested in the 4th trial series (Figure 1). *Leptocybe* appeared when the trial series was at age 5. The canopies were already inaccessible for scoring and selecting *Lepto* tolerance for the fourth generation (Figure 1). The indirect improvement of *Lepto* tolerance from the third to the fourth generation is due to the strong positive additive genetic correlation with diameter growth (Figure 5C). This study showed that the recurrent selection strategy successfully improved diameter growth and indirectly improved *Lepto* tolerance, with limited impact on *BotryoTera* tolerance.

### Proposed Selection Strategies for Diameter Growth and *Lepto* and *BotryoTera* Tolerance

*Eucalypts*, including *E. grandis*, are currently experiencing a decline, mainly due to pest and pathogen pressures for commercial deployment and breeding populations such as *Puccinia psidii* (Silva et al., 2013), *L. invasa* (da Silva et al., 2020), *T. zuluensis* (Wingfield et al., 1996; Aylward et al., 2019), and *B. dothidea* (Smith et al., 1996; Marsberg et al., 2017). This study offers opportunities to revise historical evaluation and selection strategies to improve diameter growth and *BotryoTera* and *Lepto* tolerance. Testing all these pedigree-linked *E. grandis* generations in the same space and time has highlighted the successes and challenges of traditional evaluation and selection strategies and their direct and indirect impact on economic traits over the generations as new pests and pathogens emerge. First, pests and pathogens may appear during a growth stage within a breeding cycle when trees cannot be effectively scored and selected. Second, pests and pathogens affect different parts of the tree, young leaves (early in the growth cycles), and stem (later in the growth cycles); therefore, the correct timing of scoring is crucial. Third, although present, pests and pathogens may differ in their infestation and infection severity due to many factors, leading to highly varying levels of challenge and incomplete expression of tolerance or susceptibility. Fourth, the emergence of pests and pathogens sometimes may reveal inadequacies of already established selection strategies, thereby requiring revision, as is the case for *BotryoTera*.

**FIGURE 6:**
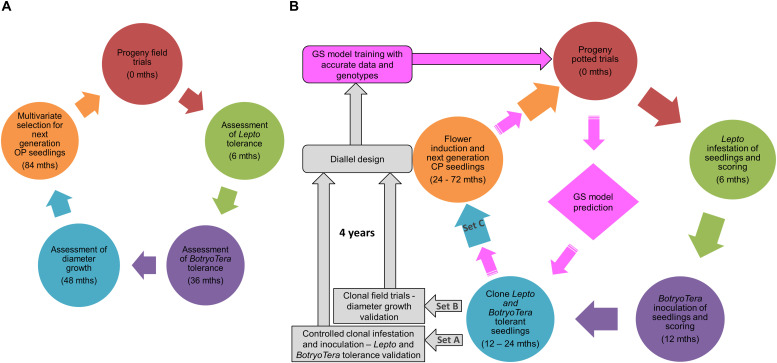
Proposed breeding strategies to improve diameter growth under pest and pathogen pressures. **(A)** Traditional field-based multivariate selection strategy whereby diameter growth (genetically correlated with *Lepto* tolerance) is the target trait. *BotryoTera* tolerance selections are made within top-ranked diameter growth families to produce open-pollinated (OP) families for the next generation. **(B)** Proposed non-field-based serial selection strategy in which *Lepto* tolerance and *BotryoTera* tolerance are scored after successive (6 and 12 months) controlled infestation and inoculations, respectively. Candidate seedlings from within these tolerant families are cloned and used for flower induction (Set C) and generation of CP families for the next generation. Another set of candidate clones is used to validate the *Lepto* and *BotryoTera* tolerance (Set A). The third set is then planted in field clonal trials for diameter growth (Set B). Accurate phenotypes from the clonal material and genome-wide genotyping of the clones create an opportunity to train a genomic selection model that can reduce (*pink arrows*) the need for expensive pest and disease phenotyping in the next generation.

A multivariate approach to deal with these challenges requires an understanding of the traits additive genetic correlations. Such a strategy would require turning over a generation in which all three traits were measured on each tree to estimate their between- and within-family breeding values. The challenge with field trials is that there are often difficulties to score pest and pathogen tolerance accurately, as discussed. Breeders may adopt a multivariate approach to primarily select for diameter growth and indirectly for *Lepto* tolerance and then only consider selecting *BotryoTera* tolerant individuals from high ranked families (Figure 6A).

Circumventing field trials and the inconsistency of pest infestations or pathogen infections, tree breeders may consider a proposed serial selection strategy with genomic selection and controlled pollination in potted trials (Figure 6B). This approach would require the integration of nursery and field phenotypes to develop a more accurate GS model. Such an approach was demonstrated in *Populus deltoids* for tree height to accelerating its breeding strategies (Alves et al., 2020). The proposed GS approach in this involves challenging potted families with *L. invasa* and scoring *Lepto* tolerance 6 months after potting and then advancing the most tolerant individuals across families for *BotryoTera* tolerance scoring at 12 months after potting. The best individuals from the top *Lepto* and *BotryoTera* tolerant families are then cloned to validate the pest and pathogen tolerance (Set A).

Meanwhile, the second set of ramets from the same clones (Set B) is planted in field trials to validate the expected correlated diameter growth response, while the third set of ramets (Set C) are subjected to flower induction to produce control-pollinated next-generation families. The clonal phenotypic data can be used together with genome-wide genotyping to train a genomic selection model for implementation (pink arrows in Figure 6B). Genomic estimated breeding values and genomic relationship matrices will inform the control pollination (diallel in the potted orchard) (Munoz et al., 2014; Li et al., 2019). This approach should increase the selection intensity and reduce the need for costly controlled pest and pathogen challenges, thereby fast-tracking clonal tests and producing next-generation control-pollinated (CP) seedlings (with breeding value predictions for all three traits) to improve gains per unit time over what can be achieved in a traditional open-pollinated (OP) field testing approach.

## Conclusion

Diameter growth and pest and pathogen tolerance are essential components of sustainable plantation forestry. Therefore, a multivariate selection approach informed by their additive genetic correlations is key to improving genetic gains in these traits simultaneously. This study shows that evaluation and selection strategies implemented for *E. grandis* over the past three generations have succeeded in improving diameter growth and indirectly *Lepto* tolerance, while limited gain was achieved for *BotryoTera* tolerance. We proposed an alternative to the traditional field-based multivariate strategy, which has many challenges mainly limited by the reliability of assessing pest infestations and pathogen infections in the field. The proposed serial genomic selection strategy involves controlled infestations with *Lepto* and inoculations with *BotryoTera* of cloned families in pots to achieve validated and accurate tolerance scores and diameter growth measurements from clonal field trials. This approach will ensure a reliable multivariate genomic selection training and development to exploit the additive genetic correlations void phenotyping challenges with field trials. The proposed genomic selection strategy, possibly via ssGBLUP (Misztal et al., 2013), would be a feasible approach to improve diameter growth and *Lepto* and *BotryoTera* tolerance in *E. grandis*.

## Data Availability Statement

The datasets presented in this study can be found in online repositories. The names of the repository/repositories and accession number(s) can be found in the article/Supplementary Material.

## Author Contributions

MM carried out the experimental design, data collection, data analysis, and drafting of this manuscript as part of his Ph.D. thesis. FI assisted with the modeling and scripts of the data analysis tools. FI, GH, and AM helped with the data interpretation and supervision. All authors have read and approved the final manuscript.

## Conflict of Interest

MM was employed by the company Mondi South Africa (Pty) Ltd. The authors declare that this study received funding from Mondi South Africa (Pty) Ltd. The funder was not involved in the study design, collection, analysis, and interpretation of data, the writing of this article, or the decision to submit it for publication.
