# Supplementary material for: Genomic Breeding for Diameter Growth and Tolerance to Leptocybe Gall Wasp and Botryosphaeria/Teratosphaeria Fungal Disease Complex in Eucalyptus grandis
Source: Front Plant Sci. 2021 Feb 26;12:638969. doi: 10.3389/fpls.2021.638969 (PMC7952757; doi:10.3389/fpls.2021.638969)
Supplement: Supplementary file 2 [file Data_Sheet_2.docx]

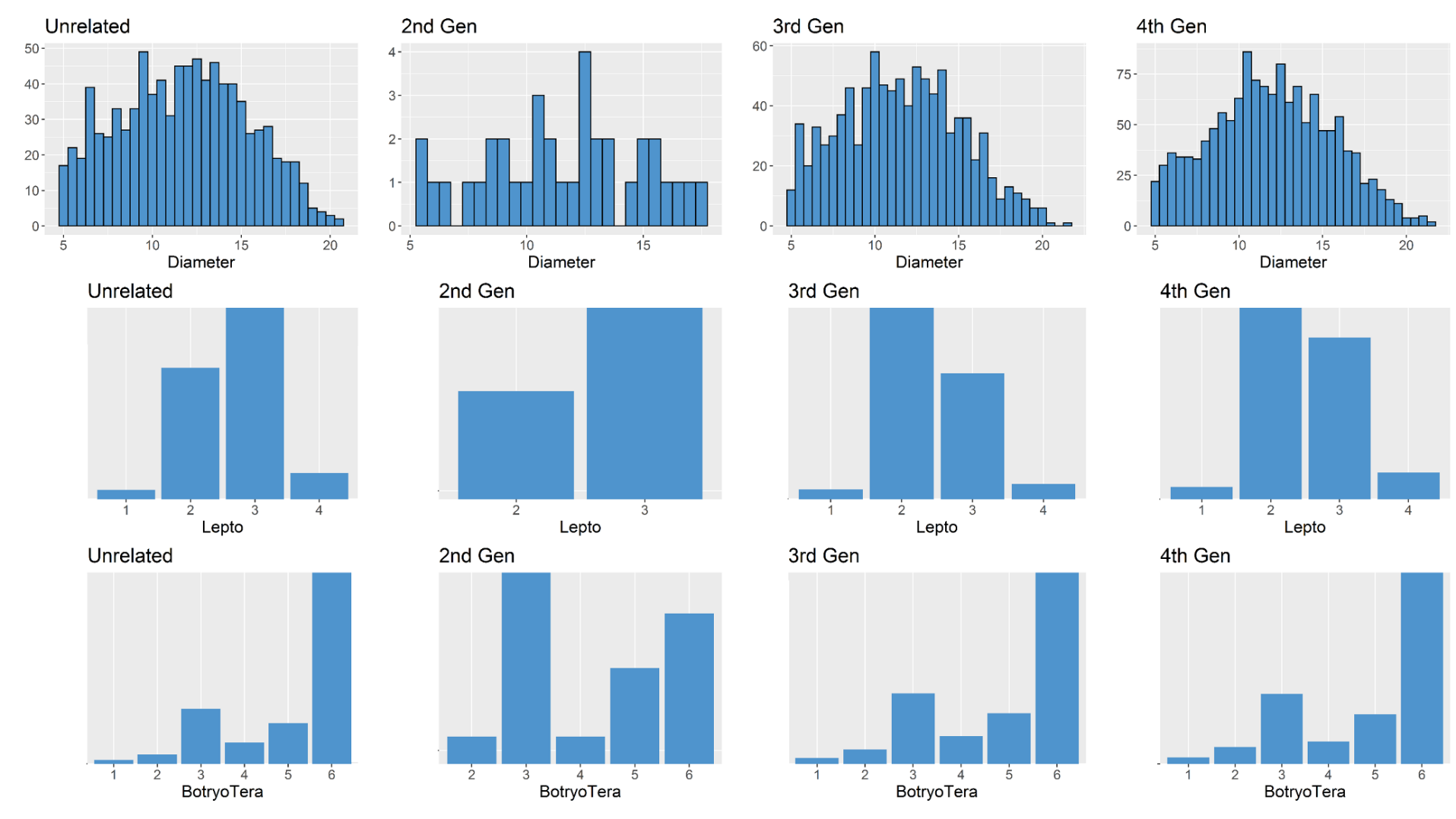
 **Supplementary FIGURE 2 |** Frequency plots across the representative pedigree linked and unrelated generations. The *top row* is the diameter growth histogram plot, the *middle row* is the histogram plot for *Lepto* incidence scores, and the *bottom row* is the *BotryoTera* incidence scores.
