# Supplementary material for: Genomic Breeding for Diameter Growth and Tolerance to Leptocybe Gall Wasp and Botryosphaeria/Teratosphaeria Fungal Disease Complex in Eucalyptus grandis
Source: Front Plant Sci. 2021 Feb 26;12:638969. doi: 10.3389/fpls.2021.638969 (PMC7952757; doi:10.3389/fpls.2021.638969)
Supplement: Supplementary file 3 [file Data_Sheet_3.docx]

**Supplementary TABLE 1 |** Environmental and trial design information of the study population. The number of families and their pedigree generations are indicated with the phenotyped and genotyped individuals.

|  |  | **Nyalazi** | **Kwambonambi** | **Mtunzini** |
| --- | --- | --- | --- | --- |
| **Site environment** | Latitude (South) | 28^ᵒ^ 12'32.01'' S | 28^ᵒ^ 38' 56.43'' S | 29^ᵒ^ 1' 52.11'' S |
|  | Longitude (East) | 32^ᵒ^ 20' 42.79'' E | 32^ᵒ^ 9' 13.81'' E | 31^ᵒ^ 39' 23.73'' E |
|  | Altitude (m) | 47 | 63 | 69 |
|  | ^a^MAP (mm) | 999 | 1196 | 1220 |
|  | ^b^MAT (C) | 21 | 21 | 21 |
|  | MAT min. (C) | 12 | 11 | 11 |
|  | MAT max. (C) | 30 | 29 | 28 |
| **Distance (km)** | Nyalazi |  | 50 | 112 |
|  | Kwambonambi |  |  | 66 |
| **Trial design** | Progeny type | Half-sib | Half-sib | Half-sib |
|  | Trial design | RCB | RCB | RCB |
|  | Replications | 15 | 15 | 15 |
|  | Plot design | Single-tree plot | Single-tree plot | Single-tree plot |
| **Pedigree** | *Unrelated families* | 33 | 33 | 31 |
|  | *2nd Gen families* | 2 | 2 |  |
|  | *3rd Gen families* | 32 | 32 | 30 |
|  | *4th Gen families* | 28 | 28 | 28 |
|  | Total families | 95 | 95 | 89 |
|  | Number of trees | 1890 | 1830 | 1680 |
| **Survival (%)** | Diameter (4yrs) | 68 | 59 | 58 |
|  | *BotryoTera* (3yrs) | 83 | 66 | 68 |
|  | *Lepto*(1.5yrs) | 89 | 80 | 78 |
| **Phenotyped individuals (*n*)** | Diameter | 1290 | 1074 | 970 |
|  | *BotryoTera* | 1573 | 1216 | 1144 |
|  | *Lepto* | 1687 | 1465 | 1311 |
| **Genotyped**  **individuals (*n*)** | Diameter | 246 | 291 | 278 |
|  | *BotryoTera* | 321 | 325 | 318 |
|  | *Lepto* | 340 | 358 | 353 |

**^a^MAP – mean annual precipitation**

**^b^MAT – mean annual temperature**
